# Development of RisObIn.Com, a Screening Tool for Risk of Childhood Obesity in the Community

**DOI:** 10.3390/nu12113288

**Published:** 2020-10-27

**Authors:** Ana Catarina Moreira, Patrícia Almeida Oliveira, Rute Borrego, Telma Nogueira, Raquel Ferreira, Daniel Virella

**Affiliations:** 1 Escola Superior de Tecnologia da Saúde de Lisboa, Instituto Politécnico de Lisboa, 1990-096 Lisboa, Portugal; patricia.aao@gmail.com (P.A.O.); rute.borrego@estesl.ipl.pt (R.B.); raquel.ferreira@cm-sintra.pt (R.F.); 2 H &TRC—Health & Technology Research Center, 1990-096 Lisboa, Portugal; 3 Faculdade de Medicina, Universidade de Lisboa, 1649-004 Lisboa, Portugal; 4 Laboratório de Nutrição, Faculdade de Medicina, Universidade de Lisboa, 1649-028 Lisboa, Portugal; telmanogueira@medicina.ulisboa.pt; 5 Instituto de Saúde Ambiental, Faculdade de Medicina, Universidade de Lisboa, 1649-028 Lisboa, Portugal; 6 Núcleo de Educação e Qualidade Alimentar, Câmara Municipal de Sintra, 2710-307 Sintra, Portugal; 7 Research Unit, Centro Hospitalar Universitário Lisboa Central, 1150-199 Lisboa, Portugal; danielvirella@chlc.min-saude.pt

**Keywords:** childhood overweight, children, risk, community, screening, tool

## Abstract

The prevalence of childhood overweight has increased considerably in the past three decades and there is evidence that childhood obesity can persist into adulthood. A simple tool to identify relevant risk factors may alert families and prevent overweight and obesity. This study aims to develop a pre-school screening tool to assess the risk of childhood obesity. Child anthropometric measurements and several risk factors for childhood obesity factors were obtained. The effect of the variables on the outcome of obesity (defined as increased anthropometry-estimated adiposity) was assessed by binary logistic regression analyses. The identified variables were submitted for expert panel validation and combined for the tool development. A total of 304 children were included. Eight items were included in the tool. A higher score of the tool indicates a greater risk for obesity in childhood with the cutoff point set at 0. The tool sensitivity for obesity was 95%, specificity was 74.4%, the positive predictive value was 37.3%, and negative predictive value was 98.9%. The Risk of childhood Obesity In the Community (RisObIn.Com) tool is proposed to be a comprehensive tool to identify children at high risk for late childhood obesity at admission to primary school. Further studies are needed to assess the performance of the tool.

## 1. Introduction

The prevalence of childhood overweight and obesity has increased considerably in the past decades, mostly in high-income countries but recently also rising in low- and middle-income countries. Although in high-income countries, a recent decrease has been observed [1], prevalence remains very high [2,3]. Obesity is defined as an abnormal fat accumulation that impairs health [4] but it has been shown that obesity defined by Body Mass Index (BMI) alone is a remarkably heterogeneous condition with varying cardiovascular and metabolic manifestations across individuals, which may differ according to age and gender [5]. This is a chronic disease that increases heavily the burden on citizens, health care systems, productivity, cities, and society and should be considered a top priority and main target to combat the increasing non-communicable diseases epidemic [6]. There is evidence that childhood obesity can persist into adulthood [7], lead to physical obesity-related complications, and affect psychological health and social and emotional well-being [8]. This emphasizes the importance of early intervention to prevent the onset of obesity in childhood. A comprehensive and proactive strategy to deal with the challenges imposed by the obesity epidemic is needed and requires the development and implementation of programs for prevention, early diagnosis, and treatment, especially in children [6]. However, interventions to reduce childhood obesity show limited effectiveness, particularly for weight-related outcomes [9].

Therefore, sustainable and effective interventions to prevent childhood obesity should target higher-risk children [10]. Obesity development involves a complex interplay between physiological environmental, psychological, social, and behavioral exposures [11,12]. There is evidence of epigenetic processes in utero that contribute to infant obesity, including DNA methylation, and gut microbiome alterations [13]. Breastfeeding is also associated with obesity protection [14,15]. Additional life course exposures include socio-economic status, food production and marketing, food insecurity, and obesogenic environments, that can promote unhealthy lifestyles. In this environment, some individuals are genetically more susceptible to develop obesity [16].

A simple tool to identify relevant obesity risk factors early in life may alert families and caretakers into positive changes, improving a child’s weight trajectory and preventing overweight and obesity. Tools to identify children at risk for obesity have been published [17,18,19]. To our best knowledge, none of these include a large diversity of parameters known to affect weight gain trajectory; the broad variables related to obesity require a more comprehensive tool.

This study aims to develop a pre-school screening tool to assess the risk for childhood obesity based on a broad spectrum of risk factors considering peri-natal, anthropometric, sociodemographic, past eating habits, current eating habits, subjective anthropometry perception, subjective eating habits perception and physical activity, and sleeping habits, at a multivariable level.

## 2. Materials and Methods

Data from the community-based participatory research Sintra Grows Healthy (SGH) [20] were used for independent analysis, as a cross-sectional with nested case-control study. The study sample comprises schoolchildren aged 6–12 years attending the first to fourth grades of six public primary schools in Sintra municipality, Portugal. Anthropometric measurements of children were assessed and a wide set of data was obtained by the application of a questionnaire to the children’s legal guardian, mainly their parents. For the purpose of the present study, an additional set of questions relevant for the study of obesity risk factors was collected by applying a second questionnaire to the children’s legal guardian. Only children for whom both these questionnaires were filled, were selected for this study. Data were collected between 2017 and 2018. Written informed consent was obtained and the safety and confidentiality of all the collected and archived data were ensured. Approval was obtained from the National Commission of Data Protection and the Ethics Commission of Lisbon Academic Medical Center.

Anthropometric measurements were obtained directly by trained members of the SGH research team, using standardized anthropometric procedures [21]. Children were barefoot and wearing minimal clothing to assess height and weight. Height was assessed using a portable stadiometer to the nearest 0.1 cm (SECA 213^®^) in the vertical position, with feet together and the head in the Frankfort plane. Weight was assessed through a portable calibrated scale (SECA Robusta 813^®^, SECA Deutschland, Hamburg, Germany), expressed up to 0.1 kg. Body Mass Index (BMI) was calculated as weight (kg) divided by height squared (cm^2^). BMI was classified according to age and gender z-scores of the World Health Organization for children aged 5 to 19 years old [22]. Waist circumference was directly measured on the skin to the nearest 0.1 cm according to the World Health Organization method with a non-extensible and flexible tape (SECA 201^®^, SECA Deutschland, Hamburg, Germany) [23]. Waist-to-Height Ratio (WtHR) was calculated as waist circumference (cm) divided by height (cm) and classified as an indicator for early health risk as ≤0.5 or >0.5 [24].

The set of questions specifically included for the present study was gathered and developed through a literature review regarding childhood obesity [11,12,16]. Children were asked to fill the questionnaire at home with their parents. To assure data confidentiality, each child was assigned a subject identification code. The questionnaires were collected by teachers and sent back to the SGH team. Data entry and revision was conducted through a standardized procedure. The information collected for the present analysis included (a) parental nationality; (b) parental level of education; (c) family type (nuclear/extended two-/one-parented); (d) number and age of siblings; (e) mean monthly income; (f) parental current employment status; (g) present parental weight and height (from which BMI was calculated and categorized into underweight, normal weight, overweight, or obese) and (h) father’s and mother’s body image perception. The caretakers were asked to recall peri-natal information: (a) maternal weight before and after pregnancy (from which BMI pre-pregnancy and adequacy of weight gain during pregnancy were calculated according to the Institute of Medicine recommendations [25]); (b) maternal tobacco consumption during pregnancy; (c) diagnosis of gestational diabetes and/or pre-eclampsia; (d) information given by the assistant physician during pregnancy on adequateness of the fetus for gestational age; and (e) the gestational age in complete weeks (to determine if the birth was pre-term or term). Information regarding anthropometric data throughout childhood was retrieved from the records in the child health bulletin: (a) birth weight and length [from which BMI was calculated through World Health Organization Anthro software for Windows, version 3.2.2. (World Health Organization—Department of Nutrition, Geneva, Switzerland), and classification into small for gestational age (SGA, < 10th percentile), appropriate for gestational age (10th–89th percentile), and large for gestational age (LGA, > 90th percentile) were obtained]; (b) weight and length at the ages of 12, 18, and 24 months and 3 and 5 years old [from which BMI z-score was calculated through the World Health Organization Anthro software for Windows, version 3.2.2. (World Health Organization—Department of Nutrition, Geneva, Switzerland)]. Overweight (including obesity) was defined according to age and sex z-scores (above 2) of the World Health Organization for children up to 5 years old [26]; since the medical visits from birth to 5-years-old may not have occurred at the exact dates selected to recall anthropometric data, the health record information closer to those ages and respective dates were collected to correctly assess z-scores. To classify anthropometric measurements at each visit, the exact age was calculated by the difference between birth date and the records visit date.

We collected data on child and family feeding patterns at two moments. We asked about breastfeeding (total and exclusive duration) and the introduction of solid foods (age, appetite, and type of meal first introduced). For present feeding pattern, we asked about: child’s appetite, the Mediterranean diet pattern index of the child (KIDMED [27]) through adapted questions, and its family (PREDIMED [28]), and one question to both child and caretakers regarding child’s intake when worried, irritated, or anxious, extracted from the from Child Eating Behaviour Questionnaire [29]. To assess the child’s and caretaker’s perception of the quantity of child usual intake, we used images of four meal plates with different portions of food (A to D, ascendingly). According to children’s height-for-age, we determined the two images closer to their recommended portion (z-score ≤ 1 corresponded to images A and B; z-score > 1 corresponded to images B and C). Image D represented an excessive food portion for any children of our sample. We compared the adequacy of the caretaker’s answers and classified them as adequate, excessive, or lower. Caretakers were asked to select an option regarding the child’s nutritional status for age between “low weight,” “adequate weight,” or “excessive weight” and compared to the child’s BMI. This perception was categorized as correct, underestimated, or overestimated.

Children and caretakers identified the child’s body figure through body image scales [30]. We compared that perception with the corresponding child’s BMI and categorized it as incorrect, relatively correct, or correct to child’s BMI, as previously done [31]. The perceptions were additionally categorized as correct, underestimated, or overestimated. Caretakers also identified their own body figures through body image scales [32]. We compared that perception with the corresponding parental BMI and categorized it as incorrect, relatively correct, or correct to child’s BMI, as previously done [33]. The perceptions were additionally categorized as correct, underestimated, or overestimated.

We questioned about the physical activity and sedentary behaviors of the child, the frequency of consumption of meals in front of a screen, and the number of hours of sleep.

Variables were grouped into eight dimensions: peri-natal, anthropometric, sociodemographic, previous eating habits, current eating habits, subjective anthropometry (perception), subjective eating habits (perception), physical activity, and hours of sleep.

### 2.1. Development of the Risk Index Tool and Scoring

As the BMI, as a single measurement of obesity, does not reflect the whole complexity of the condition [6], the European Association for the Study of Obesity proposed to improve the diagnostic criteria for obesity with the inclusion of other dimensions, including the degree of adiposity [34]. Therefore, to increase the accuracy of the outcome measure, to reflect an adiposity-based condition, a composite variable was created using BMI and WtHr. Thus, the primary outcome measure in this study is increased adiposity, defined as overweight (including obesity) with WtHr > 0.5, while in primary school. To test the effect of the factors under investigation on the primary outcome, binary logistic regression analyses were performed. Exposures were tested within the aforementioned dimensions (dependent variable: overweight (including obesity) with WtHr > 0.5; factors: all risk factors by dimension).

The regression analysis was used to identify factors associated with the primary outcome on each of the eight dimensions. The studied risk factors that showed an association with our primary outcome were presented to an expert pediatric panel (nutrition, education, nursing, pediatrician, and exercise physiology experts) for external construct validation with the purpose of developing the proposed tool: RisObIn.Com (Risk of childhood Obesity In the Community).

The most agreed risk factor variables were then combined to develop the RisObIn.Com tool. At least one item from every considered dimension was included in the score if any of the items revealed significant statistical relevance. The Beta (β) values to a decimal case were used to generate the scoring system as an indicator of the association between each variable, and 0 and 1 scores were assigned to the response option regarding their association with the outcome variable (overweight including obesity with WtHr > 0.5). As an example, on the physical activity item, a score of 0 was assigned to the response option “plays regular and programmed physical activity,” and a score of 1 to the response option “doesn’t play regular and programmed physical activity.” This score was then multiplied by 1.6 to obtain the item’s score, as β was 1.642. The final score of the RisObIn.Com tool was obtained by the sum of all item scores and a correction factor was added to obtain zero as the cut-off value.

### 2.2. Statistical Analysis

All data were checked for entry errors. Statistical analysis was done using IBM SPSS Statistics for Windows, version 26 (IBM Corp, Armonk, NY, USA). OpenEpi Version 3.01 was used to calculate confidence intervals (CI) [35]. Continuous data were checked for normal distribution using the Kolmogorov-Smirnov test and graphically by evaluating histograms and expressed as mean and standard deviation. Non-normally distributed data were expressed as median (Min-Max). Comparisons between the participants studied and those not included were made by using *t*-tests for normally distributed continuous variables, Mann-Whitney rank-sum tests for non-normally distributed continuous variables, and χ2 tests for categorical variables.

Cutoff point analysis was performed to identify the optimal value that differentiates the risk of obesity from non-risk of obesity in children. The threshold was defined by the largest distance from the diagonal line of the receiver operating characteristic (ROC) curve (sensitivity × (1 − specificity)). Using the cutoff point obtained, both sensitivity and specificity and positive and negative predictive values were calculated, with their 95% CI. All *P* values reported were based on two-sided hypotheses and compared to a significance level of 5%.

## 3. Results

### 3.1. Study Sample Characteristics

Data were collected from 593 subjects. From those, 289 had incomplete data on crucial information to proceed with the analysis (for example, sex information) and therefore were excluded. The remain 304 gathered anthropometric measurements and data regarding our set of questions and therefore were included, despite some were not complete. In the portion of not included subjects, the children’s median age was 8.0 (5.8–10.8) years old (missing 162), 7.8 (5.9–10.8) for girls, and 8.1 (5.8–10.4) for boys. The mother’s median age was 39.0 (25.0–54.0) years old (missing 62), and father’s was 41.0 (25.0–66.0) years old (missing 62). There were no significant differences between the child’s age (U = 2371.0, *p* = 0.166), mother’s age (U = 41,256.5, *p* = 0.153), child’s BMI z-score (U = 23,156.0, *p* = 0.204), mother’s (U = 38,080.0, *p* = 0.240) or father’s nationality (U = 33,481.5, *p* = 0.207), parental current employment status (U = 30,097.5, *p* = 0.308), mean monthly income (U = 33,703.5, *p* = 0.510), and father’s level of education (U = 36,689.0, *p* = 0.939) between the children included in the sample and those not included. The father’s age (U = 33,362.5, *p* = 0.021) and the mother’s level of education (U = 39,273.0, *p* = 0.035) was significantly different between the children included in the sample and those not included. The sociodemographic characteristics of the sample are presented in Table 1.

The overall prevalence estimates of underweight, normal weight, overweight, and obesity, and central adiposity are shown in Table 2. Overall, the prevalence rate was 20.7% for overweight and 9.5% for obesity. Most children (80.8%, *n* = 244) had WtHr ≤ 0.5. Combining BMI and WtHr, 16.8% [95% CI 13.0–21.4] (*n* = 51) children were overweight (including obesity) with WtHr > 0.5. Only seven children had a WtHr > 0.5 with a normal weight and four children had a WtHr ≤ 0.5 with obesity.

### 3.2. Risk Factors for Overweight (Including Obesity) with WtHr > 0.5

Risk estimation models for overweight (including obesity) with WtHr > 0.5 were explored within each dimension of variables. The significant risk factors on each of the eight dimensions are presented in Table 3 and Supplementary Table S1.

#### 3.2.1. Peri-Natal Dimension

The only variable retained in the final peri-natal dimension estimation model was maternal pre-pregnancy BMI; being classified as overweight increases, in mean, 2.6-fold the risk of overweight (including obesity) with WtHr > 0.5, and being classified as obese increases, in mean, 4.1-fold the risk of overweight (including obesity) with WtHr > 0.5.

#### 3.2.2. Anthropometric Dimension

For the anthropometric dimension, the retained variable was the BMI at 5 years old; being classified as overweight increases, in mean, 4.2-fold the risk of overweight (including obesity) with WtHr > 0.5.

#### 3.2.3. Sociodemographic Dimension

The sociodemographic variable retained in the final estimation model was paternal BMI; paternal BMI reflecting overweight decreases, in mean, 33% the risk of overweight (including obesity) with WtHr > 0.5, and paternal BMI reflecting obesity increases, in mean, 4-fold the risk of overweight (including obesity) with WtHr > 0.5.

#### 3.2.4. Past Eating Habits Dimension

For the past eating habits dimension, the only variable included in the final model was the type of meal used for solid foods introduction; if soup (rather than cereals) was the first solid food introduced, it decreases, in mean, 60% (the risk of overweight (including obesity) with WtHr > 0.5.

#### 3.2.5. Current Eating Habits Dimension

For current eating habits, the variables child’s appetite, the caretaker’s perception of the child’s intake through image, and the PREDIMED questions regarding vegetable daily intake and butter, margarine, and cream daily intake were included in the final model. The child’s appetite decreases, in mean, 83% the risk of overweight (including obesity) with WtHr > 0.5. The caretaker’s perception of the child’s intake through image increases, in mean, 1.5-fold the risk of overweight (including obesity) with WtHr > 0.5. The PREDIMED question regarding vegetable daily intake decreases, in mean, 38% (the risk of overweight (including obesity) with WtHr > 0.5, and the PREDIMED question regarding butter, margarine, and cream daily intake increases, in mean, 1.5-fold the risk of overweight (including obesity) with WtHr > 0.5.

#### 3.2.6. Subjective Anthropometry Perception Dimension

For subjective anthropometry perception, the two variables retained in the final model were (1) the adequacy of father’s own body image perception in comparison to his real BMI; and (2) the adequacy of the caretaker’s opinion regarding the child’s nutritional status in comparison to the child’s real BMI. The relatively correct adequacy of father’s own body image perception compared to real BMI increases, in mean, 4.9-fold the risk of overweight (including obesity) with WtHr > 0.5, and the correct adequacy of father’s own body image perception compared to real BMI increases, in mean, 2.6-fold the risk of overweight (including obesity) with WtHr > 0.5. Regarding the adequacy of the caretaker’s opinion regarding the child’s nutritional status compared to the child’s real BMI, correct adequacy decreases, in mean, 31.6-fold the risk of overweight (including obesity) with WtHr > 0.5.

#### 3.2.7. Subjective Eating Habits Perception Dimension

In the eating habits subjective data, the variables retained in the final estimation model were the caretaker’s perception of child’s food intake when worried, irritated, or anxious and the caretaker’s perception of the adequacy of the child’s food intake for age. The caretaker’s perception that the child’s food intake when worried, irritated, or anxious is not affected decreases, in mean, 74% the risk of overweight (including obesity) with WtHr > 0.5. The caretaker’s perception that the child’s food intake is inferior or adequate for age decreases, in mean, 92% the risk of overweight (including obesity) with WtHr > 0.5.

#### 3.2.8. Physical Activity and Sleeping Habits Dimension

For physical activity and hours of sleep, the variables included in the final model were the child’s participation in programmed sports activity, the number of sedentary hours in a weekday, the number of sedentary hours on a weekend day, and the total number of sedentary hours in a week. The child’s participation in programmed sports activity decreases, in mean, 81% the risk of overweight (including obesity) with WtHr > 0.5.

### 3.3. Scoring and Risk Index

The set of 13 variables identified for the items of the screening tool were submitted to the pediatric expert panel.

The variable father’s BMI from the sociodemographic dimension was excluded as two members of the panel did not agree with its inclusion in the tool, for lack of evidence of the variable impact in childhood obesity.

The current eating habits dimension variables for PREDIMED vegetable servings daily intake and butter, margarine, and cream servings daily intake, and the caretaker’s perception of the child’s intake through image were excluded. One member of the panel did not agree with the inclusion of the butter, margarine, and cream daily intake variable in the tool, for lack of evidence of the variable impact in childhood obesity.

The adequacy of the father’s body image perception vs. BMI from the subjective anthropometry perception was excluded as two members of the panel did not agree with its inclusion in the tool, for lack of evidence of the variable impact in childhood obesity.

For the physical activity and sleep hours dimension, only the programmed physical activity practice variable was included. The remaining were excluded due to the observed lack of measurable effect.

Eight items were included in the tool and all dimensions except the sociodemographic were included. The items and respective scores are presented in Table 4.

**Figure 1:**
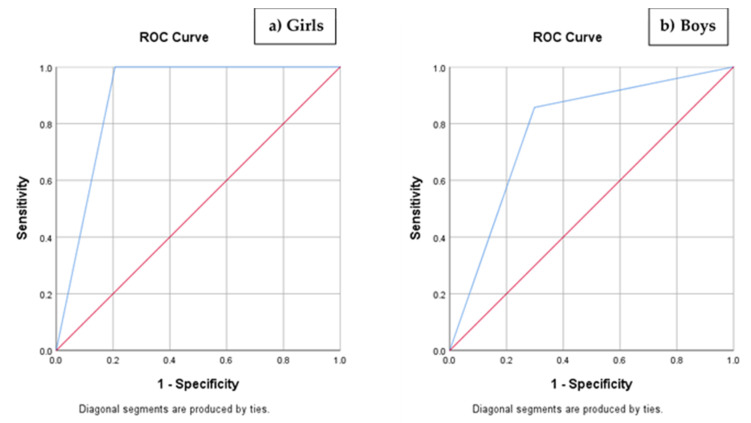
Performance of the proposed screening tool (RisObIn.Com) to identify girls (**a**) and boys (**b**) in the transition from pre-school to primary school that had overweight (including obesity) with WtHr > 0.5 while in primary school. The receiver operating characteristic (ROC) curves were calculated for the cutoff point value of zero. The area under the ROC curve was 0.897 (95%CI 0.825–0.968; *p* < 0.001) for girls; and the area under the ROC curve was 0.779 (95%CI 0.612–0.947; *p* = 0.016) for boys. CI: Confidence Interval; WtHr: Waist-to-Height Ratio.

Applying the tool items to every child who gathered responses to all the tool items (*n* = 145), the sum of the items ranged from −4.20 to 4.60. Higher values of the tool indicated a greater risk of obesity in childhood. The area under the ROC curve was 0.897 [95%CI 0.825-0.968; *p* < 0.001] for girls and 0.779 [95%CI 0.612–0.947; *p* = 0.016]—Figure 1. The uncorrected optimal cutoff point of the RisObIn.Com tool was -1, thus, a correction factor (+1) was applied to obtain a cutoff point value of zero. The tool sensitivity, based on the optimal cutoff point, was 95.0%; that is, 95.0% of children who had overweight (including obesity) with WtHr > 0.5 while on primary school, got a score greater than 0 in the transition from pre-school to primary school had overweight (including obesity) with WtHr > 0.5 while on primary school. The specificity was 74.4%, meaning that 74.4% of children who did not have overweight (including obesity) with WtHr > 0.5 while on primary school, got a score equal or less than 0 in the transition from pre-school to primary school. The positive predictive value was 37.3%, meaning that among those who had a score greater than 0, the probability of having the condition was 37.3%. The negative predictive value was 98.9%, meaning that among those who had a score equal or less than 0, the probability of not having the condition was 98.9%.

## 4. Discussion

Preschool and primary school ages are among the most critical periods for determining obesity later in life [36]. A screening tool applied at this occasion will signal cases that will benefit from general and customized intervention, improving resources to prevent child obesity.

This new proposed tool combines elements that reflect the multifactor nature of obesity: maternal BMI before pregnancy; the child’s own BMI at 5 years old; the first solid food introduced for diversification; the caretakers perception of the current appetite of the child; the parental opinion regarding the child’s intake adequacy; the child’s food intake when worried, irritated, or anxious; the parental perception of the child’s nutritional status; and the regular practice of programmed physical activities. Through this weighted combination, RisObIn.Com provides a score that estimates the risk of obesity through school age.

To the best of our knowledge, this is the first screening tool to assess the risk of obesity in children at the entrance of primary school that includes a set of parameters from different dimensions and specific periods, acknowledged to affect weight gain trajectory. The development of this tool contrasts with other existing tools, which selected items through the opinion of experts [17] or literature review [37], focusing only on a specific period of childhood, such as the peri-natal period [18] or the present moment of assessment [19]; others attempted to associate a large number of variables to childhood obesity risk through data mining [38]. The inclusion of all these dimensions can justify the large areas under the ROC curve obtained by RisObIn.Com, larger than other tools [18,19,38]. We observed a difference between boys and girls regarding the ROC findings considering the sex variable, but we consider that it is not possible to infer this difference is maintained when the tool is applied in samples differ in dimension or population characteristics. RisObIn.Com achieves 95% sensitivity and 74.4% specificity despite the tool having been developed using a smaller sample than other studies.

### 4.1. Peri-Natal Dimension

Maternal pre-pregnancy BMI was the only variable retained in the final estimation model from the peri-natal dimension set. This variable has been shown to be positively associated with infant adiposity, as well as childhood obesity and overweight [12]. A recent meta-analysis identified significantly higher odds for childhood obesity with higher pre-pregnancy maternal BMI, 89% for the offspring of overweight women before pregnancy and 264% for those who were obese before pregnancy [39].

### 4.2. Anthropometric Dimension

On the anthropometric dimension, the retained variable was the BMI at 5 years of age. The growth patterns of BMI during childhood, particularly during critical periods, are closely related to adult obesity risk [40]. The second physiological rise in BMI occurs, in general, between 3 and 7 years [41]. Pei et al., in 2013, on the German birth cohorts GINIplus—The German Infant Study on the Influence of Nutrition Intervention plus Air pollution and Genetics on Allergy Development, and LISAplus—Influence of Life-style factors on Development of the Immune System and Allergies in East and West Germany plus Air Pollution and Genetics on Allergy Development, also found that BMI at 60–64 months of age was significantly associated with overweight at the age of 10 years [42]. Children with higher BMI at 5 years of age probably have already experienced an adiposity rebound, and early age at adiposity rebound is known to be a risk factor for later obesity [43].

### 4.3. Previous Eating Habits Dimension

Evidence shows that breastfeeding is a protective factor for obesity [14,15]. However, in this study, regarding previous eating habits, we found that only the type of first complementary solid food introduced was associated with overweight (including obesity) with WtHr > 0.5 while in primary school. Recent data does not support the hypothesis that the quality of complementary foods has a direct effect on the risk for later obesity [44]. Soup can be prepared using many different vegetables as ingredients; the quality and quantity of vegetables and fat used have a strong influence on its nutritional value. By opposition, infant cereals do not allow nutrient composition modifications, thus having a more constant energy value. In countries [45] such as Portugal, the traditional recommendation of the first complementary food to be introduced is infant cereals [46]. One can only speculate that, in infants that present a higher BMI or rapid weight gain [47], health professionals will recommend that the first food to introduce should be soup, since it allows to manipulate the amount of vegetables and fat content, in the attempt to reduce energy intake.

### 4.4. Current Eating Habits Dimension

In the current eating habits dimension, the parameters retained in the final model were the child’s appetite and the caretaker’s perception of high, low, or adequate appetite of the child. These measures relate to appetite and reflect self-regulation of energy intake. Biological regulation of appetite is very complex, engaging a number of tissues, organs, hormones, and neural circuits with several feedback pathways between the brain and peripheral tissues [48]. These mechanisms can be influenced and modulated by several factors, beyond the aim of the development of a screening tool. Adequate nutritional status children eat smaller portions at lunch/dinner and may eat more snacks throughout the day; the energy of those snacks is usually greater than the energy of lunch or dinner meals [49].

When parents perceive their child as having increased appetite, they may try to implement restrictive feeding practices. These practices can increase the preoccupation of the child with food and affect eating behaviors, eventually leading to paradoxical weight gain [50]. As both extremes of feeding practices could shape children’s relation to food, we cannot exclude that, in the past, parents may have forced their children to eat after they are satisfied, promoting a dysregulation on this equilibrium, once the tendency to encourage children to clear their plate is reported to be associated with obesity [51].

The variables from the PREDIMED index, collected in the SGH study, while explored in the analysis, were excluded, because this Mediterranean diet assessment tool is not yet validated for Portugal; therefore, it may not be adapted to Portuguese food habits. The caretaker’s perception of the child’s intake through image was also excluded. We presented caretakers a set of four images of a lunch/dinner plate. By presenting an even number of options instead of an odd number, we avoid the tendency for the selection of the central option, improving the reliability of the answer. However, since the need to use images will be a challenge for the application of this tool and the removal of the item had a small effect in the final model, it was removed.

### 4.5. Subjective Perception of Anthropometry Dimension

Regarding the subjective perception of anthropometry, this study found a high proportion of parents that misclassified the nutritional status of their child. It has been speculated that the high prevalence of overweight and obesity in children in the last decades can influence the misinterpretation of the child’s normal weight [52], leading to the perception of “normal” weight despite the BMI indication of overweight. If parents cannot recognize their child as overweight, they will not act to change behaviors and the situation can exacerbate, leading to obesity.

### 4.6. Subjective Perception of Eating Habits Dimension

Adequate dietary habits are important for health throughout life, but particularly during childhood, considering that the dietary habits during this specific period are potentially perpetuated into adulthood [53]. In the dimension related to the subjective perception of eating habits, the variables retained in the final estimation model were the caretaker’s perception of the child’s food intake while worried, irritated, or anxious and the caretaker’s perception of the adequacy for age of the child’s usual food intake. Previous studies have shown psychopathology to be associated with overweight in children [54,55]; validity data indicate that children as young as 4 years old can report on their own anxiety symptoms [56]. In reaction to anxiety, emotional eating acts as a biological response that provides temporary feelings of gratification/satisfaction [57]. The intervention approach for children with anxiety symptoms and emotional eating should be adapted to address the negative emotions underlying eating behaviors as well as teaching healthy coping strategies for these emotions [58]. Parents and their own perception of the child’s dietary habits is one of the most important factors for the dietary habits of children [59]. Childhood obesity experts recommend that childhood overweight prevention should focus on parents, according to the growing evidence of the role of parental practices and family environment in promoting effective changes [11,12].

### 4.7. Physical Activity and Sleeping Habits Dimension

For the dimension concerning physical activity and sleeping habits, the variables included in the final model were the child’s participation in programmed sports activity. The levels of physical inactivity are rising in many countries with major implications for general health and the prevalence of non-communicable diseases, such as obesity. The association had already been identified in a similar population living in Portugal’s capital (Greater Lisbon) [60]. A recent multinational cross-sectional study demonstrated that low levels of moderate-to-vigorous physical activity or high sedentary levels during weekdays and weekends were associated with higher odds of obesity in 9–11-year-old children in 12 countries [61].

The RisObIn.Com tool seems to be a comprehensive tool to identify, at school entrance, 5-to-6-year-old children at higher risk for late childhood obesity. It was conceived to be applied either by the parents or by teachers or school health professionals, such as a school nurse, school nutritionist, or school social worker, with parental feedback and the child’s health bulletin for easier recall of mother pre-pregnancy BMI and anthropometric measures at the age of 5 years. The tool carries a small and simple set of instructions for its effective use. A web-based tool allows a quick, simple, and automated form of application. RisObIn.Com is hosted in Health & Technology Research Center webpage available at https://htrc.estesl.ipl.pt/risobin-com/.

The synergy between the authors of this study and the SGH research team benefited from the logistics associated with data collection and allowed the enhancement and efficiency of resources. The methodology included direct anthropometric measurements, assessment of a set of variables identified as influent in obesity development (from peri-natal to the present moment, including sociodemographic, anthropometric, past and current eating habits, subjective anthropometry, and eating habits perception, and physical activity and hours of sleep) and had the endorsement of an expert panel group composed by skilled professionals from different areas. As a positive asset, the selected outcome measure reflects adiposity by the cumulative outcome of overweight (including obesity) with WtHr > 0.5, which allows both a better characterization of the nutritional status and better accuracy. On the other hand, the mixed design of case-control study nested on a cross-sectional study is a limitation, due to recollection bias, and causal inferences to be made, just epidemiologic and statistic associations. Another source of bias was related to maternal weight before and after pregnancy that was reported and not measured. The local nature of the sample and the differences observed between the children included and not included in the study do not allow immediate generalization of the findings to other populations; therefore, external validation of the screening tool and the study of its performance in different samples is required. Eventual ethnic differences were not explored, due to national ethical and legal restrictions related to data protection. The effective sample size was smaller than expected and it probably affected the ability to identify more exposure variables significantly associated with the study outcome; a larger sample would probably have improved the observed results. Longitudinal data analysis would allow evaluating the tool’s ability to predict BMI change over time.

The RisObIn.Com screening tool is proposed to be routinely used by teachers and other school personnel with the participation of parents or caretakers for early identification of children who might benefit from preventive actions, but its use could also be extended to health care professionals such as nurses, family physicians, or pediatricians. The RisObIn.Com screening tool is a simple and inexpensive tool that can provide an evaluation of the risk factors for pediatric obesity and may identify those in need for healthy lifestyle changes.

## 5. Conclusions

The RisObIn.Com tool is proposed to be a comprehensive tool to identify children at high risk for late childhood obesity at admission to primary school, by the age of 5 to 6 years old. Further studies are needed to assess the external validity and the generalization of the findings, as well as to confirm both the performance of this tool to identify children with obesity risk at admission into primary school and the effect of the subsequent intervention to prevent obesity in children.

## Figures and Tables

**Table 1:** Sociodemographic characteristics of the study sample.

Sample Characteristics			*n*	%	Girls	Boys
					*n*	*n*
Child	Age *n* = 300		Median 8.0 (5.9–10.2) years old			
	Sex *n* = 304	Girls	148	48.7	n/a	n/a
		Boys	156	51.3	n/a	n/a
	Body Mass Index *n* = 304	Underweight	4	1.3	1	3
		Normal weight	208	68.4	95	113
		Overweight	63	20.7	38	25
		Obesity	29	9.5	14	15
Caretakers	Mother’s age *n* = 300		Median 38.0 (26.0–52.0)			
	Father’s age *n* = 280		Median 40.0 (26.0–63.0)			
	Mother’s Body Mass Index *n* = 288	Underweight	9	3.1	3	6
		Normal weight	165	57.3	82	83
		Overweight	84	29.2	43	41
		Obesity	30	10.4	14	16
	Father’s Body Mass Index *n* = 275	Underweight	0	0	0	0
		Normal weight	104	37.8	57	47
		Overweight	132	48.0	55	77
		Obesity	39	14.2	22	17
	Mother’s nationality *n* = 290	Portuguese	270	93.1	137	133
		Non-Portuguese	20	6.9	9	11
	Father’s nationality *n* = 277	Portuguese	265	95.7	131	134
		Non-Portuguese	12	4.3	6	6
	Mother’s education *n* = 297	Basic education or lower	62	20.9	32	30
		Higher secondary education or a professional course	119	40.1	68	51
		Graduation or bachelor’s degree	99	33.3	44	55
		Master or doctoral degree	17	5.7	3	14
	Father’s education *n* = 280	Basic education or lower	91	32.5	48	43
		Higher secondary education or a professional course	129	46.1	59	70
		Graduation or bachelor’s degree	46	16.4	22	24
		Master or doctoral degree	14	5.0	6	8
	Professional situation *n* = 263	Both parents are employed	209	79.5	108	101
		Only one parent is employed	44	16.7	21	23
		Both parents are jobless	10	3.8	3	7
	Mean monthly household income *n* = 271	Less than €500	9	3.32	4	5
		€500–€1000	80	29.52	40	40
		€1000–€1500	80	29.52	36	44
		€1500–€2000	52	19.18	28	24
		€2000–€3000	41	15.12	17	24
		Above €3000	9	3.32	3	6

n/a—not applicable.

**Table 2:** Characterization of anthropometric measurements and calculated indexes.

		Total				Girls				Boys			
		*n*	%	95% Confidence Interval		*n*	%	95% Confidence Interval		*n*	%	95% Confidence Interval	
				Lower Level	Upper Level			Lower Level	Upper Level			Lower Level	Upper Level
**Body Mass Index z-Score**		Median 0.36 (−2.77–3.89)											
**Body Mass Index Class**	**Underweight**	4	1.3	0.5	3.3	1	0.68	0.1	3.7	3	1.92	0.7	5.5
	**Normal Weight**	208	68.4	62.9	73.4	95	64.19	56.2	71.5	113	72.44	64.9	78.8
	**Overweight**	63	20.7	16.6	25.6	38	25.68	19.3	33.3	25	16.02	11.1	22.6
	**Obesity**	29	9.5	6.7	13.4	14	9.45	5.7	15.3	15	9.62	5.9	15.3
	***n* Total**	304	100.0	-	-	148	100.0	-	-	156	100.0	-	-
**Waist Circumference**		Median 58.5 (37.5–91.5)											
**Waist-to-Height ratio**		Median 0.46 (0.34–0.66)											
	**≤0.5**	244	80.8	75.9	84.8	110	74.8	67.2	81.2	134	86.5	80.2	90.9
	**>0.5**	58	19.2	15.5	24.4	37	25.2	18.9	32.8	21	13.5	9.0	19.8
	***n* Total**	302	100.0	-	-	147	100.0	-	-	155	100.0	-	-
**Overweight (Including Obesity) with Waist-to-Height ratio**	**>0.5**	51	16.8	13.0	21.4	31	20.9	15.3	28.4	20	12.8	8.5	18.9
	***n* Total**	304	-	-	-	148	-	-	-	156	-	-	-

**Table 3:** Variables retained in the statistical models.

Dimension	Variables			Exp(β) (95% Confidence Interval)	*p*-Value
Peri-natal	Pre-pregnancy Body Mass Index		Pre-pregnancy Body Mass Index of Overweight	2.591 (1.164–5.766)	0.020
			Pre-pregnancy Body Mass Index of Obesity	4.145 (0.925–8.570)	0.063
	Body Mass Index z-score 5-years-old			4.159 (2.404–8.497)	0.000
	Paternal Body Mass Index		Paternal Body Mass Index of Overweight	0.772 (0.252–2.364)	0.650
			Paternal Body Mass Index of Obesity	4.041 (1.271–12.844)	0.018
	Type of meal introduced in solid food introduction		Soup	0.401 (0.176–0.914)	0.030
Current eating habits	Child’s appetite	Would eat only with insistence or frequently would not eat in totality; Would eat all and be satisfied		0.174 (0.050–0.603)	0.006
	Caretaker’s perception of child’s intake through image			1.489 (0.985–2.249)	0.059
	Family Mediterranean pattern (PREDIMED)		How many vegetable servings do you consume per day?	0.624 (0.389–0.999)	0.050
			How many servings of butter, margarine, or cream do you consume per day?	1.535 (0.976–2.413)	0.063
Subjective anthropometry perception	Adequacy of father’s body image perception vs. actual Body Mass Index		Relatively correct	4.902 (1.116–21.536)	0.035
			Correct	2.597 (0.634–10.643)	0.185
	Adequacy of the caretaker’s opinion on the child’s nutritional status vs. child’s Body Mass Index		Relatively correct	3.483 (0.882–13.753)	0.075
			Correct	31.605 (6.055–164.951)	0.000
Subjective eating habits perception	Caretaker’s perception regarding child’s intake when anxious		No	0.260 (0.056–1.204)	0.085
	Caretaker’s perception of the adequacy of the child’s food intake for age		Inferior or adequate	0.083 (0.024–0.286)	0.000
Physical activity and hours of sleep	Child’s participation in programmed sport activity		Yes	0.194 (0.052–0.724)	0.015

**Table 4:** Items included in the Risk of childhood Obesity in the Community (RisObIn.Com) tool, categorization, and scoring.

Dimension	Item	Response Options and Scoring	Scoring
Anthropometric	Body Mass Index at 5 years old	0—Overweight	−2.3 (*p* = 0.031)
		1—Underweight, Normal weight	
Peri-natal	Mother’s pre-pregnancy Body Mass Index	0—Obesity	−1.0 (*p* = 0.063)
		1—Underweight, Normal weight, Overweight	
Current eating habits	Child’s appetite	0—Would eat only with insistence or frequently would not eat in totality; Would eat all and be satisfied	1.5 (*p* = 0.015)
		1—Would eat more than what is offered	
Previous eating habits	Type of meal introduced in solid food introduction	0—Soup	−0.9 (*p* = 0.030)
		1—Infant cereal	
Subjective eating habits perception	Caretaker’s perception of the adequacy of the child’s food intake for age	0—Less than adequate; Adequate	2.5 (*p* = 0.000)
		1—More than adequate	
	Caretaker’s perception regarding the child’s higher intake when worried, irritated, or anxious	0—No	1.4 (*p* = 0.085)
		1—Yes	
Subjective anthropometry perception	Adequacy of the caretaker’s opinion on the child’s nutritional status vs. child’s Body Mass Index	0—Correct	3.4 (*p* = 0.000)
		1—Incorrect	
Physical activity and hours of sleep	Child’s participation in programmed sport activity	0—Yes	1.6 (*p* = 0.015)
		1—No	

## Supplementary Materials

The following are available online at https://www.mdpi.com/2072-6643/12/11/3288/s1, Table S1: Variables in the statistical model.
