# Supplementary material for: Development of RisObIn.Com, a Screening Tool for Risk of Childhood Obesity in the Community
Source: Nutrients. 2020 Oct 27;12(11):3288. doi: 10.3390/nu12113288 (PMC7692101; doi:10.3390/nu12113288)
Supplement: Supplementary file 1 [file nutrients-12-03288-s001.pdf]

nutrients

Table S1. Variables in the statistical model

<table>
<tr><th>Dimension</th><th>n included in the analysis</th><th colspan="2">Variables</th><th>Exp(β) [95% Confidence Interval]</th><th>p-value</th></tr>
<tr><td rowspan="12">Peri-natal</td><td rowspan="12">226</td><td rowspan="2">Pre-pregnancy BMI</td><td>Pre-pregnancy BMI of Overweight</td><td>2.591 [1.164-5.766]</td><td>0.020</td></tr>
<tr><td>Pre-pregnancy BMI of Obesity</td><td>4.145 [0.925-8.570]</td><td>0.063</td></tr>
<tr><td colspan="2">Adequacy of weight gain during pregnancy based on Institute of Medicine recommendations</td><td></td><td></td></tr>
<tr><td colspan="2">Gestational weeks: term or preterm pregnancy</td><td></td><td></td></tr>
<tr><td colspan="2">Diagnosis of gestational diabetes</td><td></td><td></td></tr>
<tr><td colspan="2">Diagnosis of pre-eclampsia</td><td></td><td></td></tr>
<tr><td colspan="2">Smoking during pregnancy</td><td></td><td></td></tr>
<tr><td colspan="2">Information during pregnancy on child SGA</td><td></td><td></td></tr>
<tr><td colspan="2">Information during pregnancy on child LGA</td><td></td><td></td></tr>
<tr><td colspan="2">Birth BMI</td><td></td><td></td></tr>
<tr><td colspan="2">Small or Large for gestational age</td><td></td><td></td></tr>
<tr><td colspan="2"></td><td></td><td></td></tr>
<tr><td rowspan="5">Anthropometric</td><td rowspan="5">141</td><td colspan="2">BMI z-score 1-year-old</td><td></td><td></td></tr>
<tr><td colspan="2">BMI z-score 18-months-old</td><td></td><td></td></tr>
<tr><td colspan="2">BMI z-score 2-years-old</td><td></td><td></td></tr>
<tr><td colspan="2">BMI z-score 3-years-old</td><td></td><td></td></tr>
<tr><td colspan="2">BMI z-score 5-years-old</td><td>4.159 [2.404-8.497]</td><td>0.000</td></tr>
<tr><td rowspan="12">Sociodemographic</td><td rowspan="12">138</td><td colspan="2">Family type</td><td></td><td></td></tr>
<tr><td colspan="2">Number of siblings</td><td></td><td></td></tr>
<tr><td colspan="2">Place of the sibling (only child, older or younger sibling)</td><td></td><td></td></tr>
<tr><td colspan="2">Maternal nationality</td><td></td><td></td></tr>
<tr><td colspan="2">Paternal nationality</td><td></td><td></td></tr>
<tr><td colspan="2">Maternal level of education</td><td></td><td></td></tr>
<tr><td colspan="2">Paternal level of education</td><td></td><td></td></tr>
<tr><td colspan="2">Mean monthly income</td><td></td><td></td></tr>
<tr><td colspan="2">Parents current employment status</td><td></td><td></td></tr>
<tr><td colspan="2">Maternal BMI</td><td></td><td></td></tr>
<tr><td rowspan="2">Paternal BMI</td><td>Paternal BMI of Overweight</td><td>0.772 [0.252-2.364]</td><td>0.650</td></tr>
<tr><td>Paternal BMI of Obesity</td><td>4.041 [1.271-12.844]</td><td>0.018</td></tr>
<tr><td rowspan="3">Previous eating habits</td><td rowspan="3">243</td><td colspan="2">Exclusive breastfeeding duration</td><td></td><td></td></tr>
<tr><td colspan="2">Total breastfeeding duration</td><td></td><td></td></tr>
<tr><td colspan="2">Age of solid food introduction</td><td></td><td></td></tr>
</table>

		**Type of meal introduced in solid food introduction**	**Soup**	**0.401 [0.176-0.914]**	**0.030**
		Appetite during solid food introduction			
Current eating habits	199	**Child appetite**		**0.174 [0.050-0.603]**	**0.006**
		**Caretakers' perception of child intake through image**		**1.489 [0.985-2.249]**	**0.059**
		Child perception of intake through image			
			Take a fruit or fruit juice every day		
			Has a second fruit every day		
			Has fresh or cooked vegetables regularly once a day		
			Has fresh or cooked vegetables more than once a day		
			How many times a week you go to fast-food restaurants or eat pre-cooked meals?		
			For breakfast, most days of the week you eat		
			How many times a week you eat sweets or candy?		
			Total Score of the full KIDMED index		
		How many times a week you drink soda?			
		**Family Mediterranean pattern (PREDIMED)**	Do you use olive oil as the main culinary fat?		
			How much olive oil do you consume in a given day		
			**How many vegetable servings do you**	**0.624 [0.389-0.999]**	**0.050**

			**consume per day?**		
			How many fruit units do you consume per day?		
			How many servings of red meat, hamburger, or meat products do you consume per day?		
			**How many servings of butter, margarine, or cream do you consume per day?**	**1.535 [0.976-2.413]**	**0.063**
			How many sweet or carbonated beverages do you drink per day?		
			How much wine do you drink per week?		
			How many servings of legumes do you consume per week?		
			How many servings of fish or shellfish do you consume per week?		
			How many times per week do you consume commercial sweets or pastries?		
			How many servings of nuts (including peanuts) do you consume per week?		
			Do you preferentially		

			consume chicken, turkey, or rabbit meat instead of veal, pork, hamburger or sausage?		
			How many times per week do you consume vegetables, pasta, rice, or other dishes seasoned with sofrito?		
Subjective anthropometry perception	251	**Adequacy of fathers' body image perception vs. actual BMI**	**Relatively correct**	**4.902 [1.116-21.536]**	**0.035**
			**Correct**	**2.597 [0.634-10.643]**	**0.185**
		Adequacy of mother's body image perception vs. actual BMI			
		**Adequacy of the caretaker's opinion on the child nutritional status vs. child BMI**	**Relatively correct**	**3.483 [0.882-13.753]**	**0.075**
			**Correct**	**31.605 [6.055-164.951]**	**0.000**
		Adequacy of the caretaker's body image perception of the child vs. child BMI			
		Adequacy of the child body image perception vs. actual BMI			
Subjective eating habits perception	256	**Caretaker's perception regarding child intake when anxious**	**No**	**0.260 [0.056-1.204]**	**0.085**
		Child perception regarding intake when anxious			
		Difference between child perception of intake through image and caretakers' perception of the child intake through image			
		**Caretakers' perception of the adequacy of the child food intake for age**	**Inferior or adequate**	**0.083 [0.024-0.286]**	**0.000**
		Adequacy of food intake for height			
Physical activity and hours of sleep	91	**Child participation in programmed sport activity**	**Yes**	**0.194 [0.052-0.724]**	**0.015**
		Consumption of meals in front of a screen			
		Consumption of meals in front of a screen: How many times a week?			

		Hours of sleep, weekdays		
		Hours of sleep, weekend days		
		Hours of sleep, mean		

© 2020 by the authors. Licensee MDPI, Basel, Switzerland. This article is an open access article distributed under the terms and conditions of the Creative Commons Attribution (CC BY) license (http://creativecommons.org/licenses/by/4.0/).
